# The Polycomb group protein RING1B is overexpressed in ductal breast carcinoma and is required to sustain FAK steady state levels in breast cancer epithelial cells

**DOI:** 10.18632/oncotarget.1779

**Published:** 2014-02-14

**Authors:** Almudena Bosch, Konstantina Panoutsopoulou, Josep Maria Corominas, Ramón Gimeno, Gema Moreno-Bueno, Juan Martín-Caballero, Saleta Morales, Tania Lobato, Carles Martínez-Romero, Eduardo F. Farias, Xavier Mayol, Amparo Cano, Inmaculada Hernández-Muáoz

**Affiliations:** ^1^ Cancer Research Program. IMIM (Institut Hospital del Mar d'Investigacions Mèdiques). Barcelona. Spain; ^2^ Departamento de Bioquímica. Facultad de Medicina. Universidad Autónoma. Instituto de Investigaciones Biomédicas “Alberto Sols”, CSIC-UAM. Instituto de Investigación Sanitaria La Paz. Madrid. Spain; ^3^ Laboratory Animal Units. Parc de Recerca Biomèdica de Barcelona. Spain; ^4^ Servei de Patologia, Hospital del Mar. Barcelona. Spain; ^5^ Servei de Inmunologia. Hospital del Mar. Barcelona. Spain; ^6^ Current address: Department of Medicine. Mount Sinai School of Medicine. New York. USA

**Keywords:** Ring1B, ductal breast carcinoma, Fak, p63, mammary epithelial cell

## Abstract

In early stages of metastasis malignant cells must acquire phenotypic changes to enhance their migratory behavior and their ability to breach the matrix surrounding tumors and blood vessel walls. Epigenetic regulation of gene expression allows the acquisition of these features that, once tumoral cells have escape from the primary tumor, can be reverted. Here we report that the expression of the Polycomb epigenetic repressor Ring1B is enhanced in tumoral cells that invade the stroma in human ductal breast carcinoma and its expression is coincident with that of Fak in these tumors. Ring1B knockdown in breast cancer cell lines revealed that Ring1B is required to sustain Fak expression in basal conditions as well as in Tgfβ-treated cells. Functionally, endogenous Ring1B is required for cell migration and invasion in vitro and for in vivo invasion of the mammary fat pad by tumoral cells. Finally we identify p63 as a target of Ring1B to regulate Fak expression: Ring1B depletion results in enhanced p63 expression, which in turns represses Fak expression. Importantly, Fak downregulation upon Ring1B depletion is dependent on p63 expression. Our findings provide new insights in the biology of the breast carcinoma and open new avenues for breast cancer prognosis and therapy.

## INTRODUCTION

Breast cancer is a heterogeneous disease, ranging from premalignant hyperplasia to invasive and metastatic carcinomas. Metastatic dissemination of tumoral cells contributes to more than 90% of mortality in breast cancer and is the second leading cause of cancer related mortalities in women. In addition to well characterized genetic alterations, breast cancer also appears to exhibit specific epigenetic modifications [1]. Epigenetic changes in the tumoral cells may promote metastasis by selecting those cells with enhanced invasiveness and colonizing faculties. Indeed, aberrant expression of a number of histone modifying enzymes has been correlated with breast cancer prognosis [2-5].

The Polycomb family of epigenetic proteins comprises transcriptional repressors that are often misregulated in several types of human cancer, impinging on tumor proliferation, immortalization and metastasis. In mammals, two main biochemically and functionally distinct Polycomb core complexes have been identified: Polycomb repressive complex 2 and 1 (PRC2 and PRC1, respectively) [6]. The PRC2 core complex contains the histone methyltransferase Ezh2, which catalyzes the trimethylation of histone H3 at lysine 27 (H3K27me3), a transcriptionally repressive histone mark [7]. The H3K27me3 is the docking site for proteins of the PRC1 complex harboring a chromobox domain, although functional PRC1 complexes can also be recruited in the absence of PRC2 [8,9]. The catalytic activity of PRC1 relies on Ring1B, which acts as ubiquitin E3 ligase towards histone H2A at lysine 119 [10]. Proteomic profiling of the family of PRC1 complexes revealed that, in mammals, distinct and heterogeneous PRC1 complexes that exert specific functions could be formed [11]. All PRC1 complexes contain Ring1B, that forms functionally different PRC1 complex through its interaction with different combinations of the Drosophila PSC orthologs (Mel-18, Bmi1, or NSPC1), PH orthologs (Phc1, Phc2, or Phc3) and PC orthologs (Cbx2, Cbx4, Cbx6, Cbx7, or Cbx8) [12]. PRC1 functional complexity is highlighted by the fact that Ring1B knockout mice are early embryonic lethal, whereas Mel-18, Cbx2, or Bmi1 mutant mice are born but display distinct homeotic phenotypes [13-16].

Up to now, the role of Polycomb in breast cancer has been mainly addressed to investigate the expression or the function of two Polycomb proteins, EZH2 and BMI1 (PRC2 and PRC1 complexes, respectively). Indeed, overexpression of EZH2 was shown to correlate with breast cancer aggressiveness and poor patient prognosis [2,4] and the PRC2 complex is up-regulated in breast cancer lymph node metastasis compared to primary tumors [17]. On the contrary, EZH2 knockdown in highly aggressive MDA-MB-231 cells decreased the metastatic burden and reduced the invasiveness of breast cancer cells at the metastatic site [18]. BMI1 overexpression often correlates with poorer prognosis and treatment failure in different epithelial tumors [19-22]. However, the relationship between BMI1 expression and prognosis in breast cancer remains controversial: although it has been reported that BMI1 is overexpressed in advanced stages of breast cancer [23], BMI1 expression has been linked to good outcome in breast cancer [4].

Our group has previously demonstrated that Ring1B expression is significantly and persistently up-regulated in high-grade pancreatic intraepithelial neoplasia and in pancreatic ductal adenocarcinoma, but not in early stages of the neoplasia or in precursor lesions for pancreatic cancer [24]. Therefore, we postulated that Ring1B could be required for essential properties of the tumoral cell in invasive stages of epithelial tumors. In this study we sought to explore the role of Ring1B in breast cancer. We here describe that Ring1B displays a coincident expression with Fak in human invasive ductal breast carcinomas and ectopic Ring1B is able to enhance Fak expression and cell migration *in vitro*. Accordingly, endogenous Ring1B is required for proper Fak expression in mammary epithelial tumor cells *in vitro*. Functionally, Ring1B expression is required for *in vitro* cell migration and invasion and for *in vivo* MDA-MB-231 invasion of the mammary stroma of the murine orthotopic xenograft host. Finally, in an attempt to identify the molecular mechanism underlying this phenomenon, we report that maintenance of Fak steady state levels relies on p63 repression by endogenous Ring1B.

## RESULTS

### Ring1B is overexpressed in the invading cells of ductal breast carcinoma

**Figure 1:**
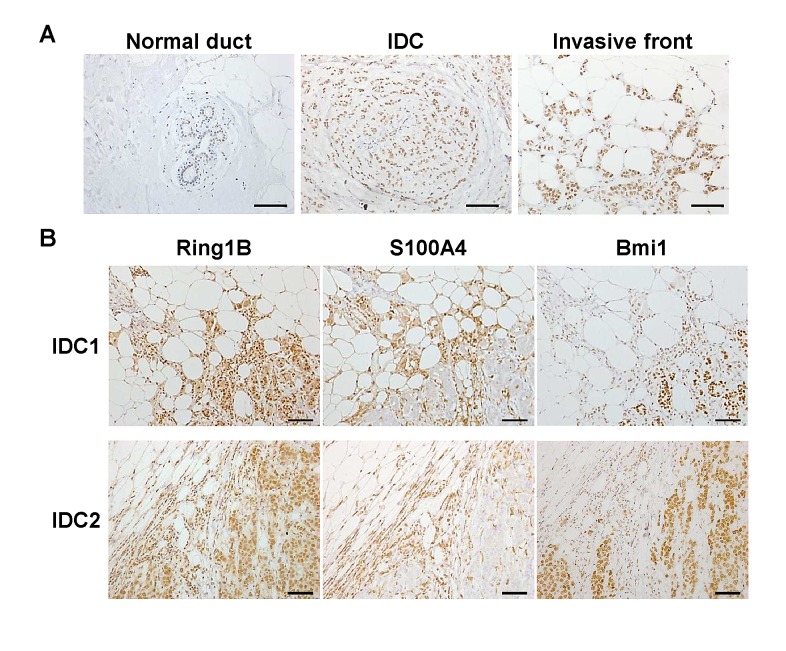
Ring1B expression in invasive ductal breast carcinoma A. Staining for Ring1B is weak in cells of adjacent normal human mammary ducts (picture on the left), medium in invasive ductal carcinoma cells (middle) and strong in carcinoma cells invading mammary fat (right) within the same tumor tissue. Sections were counterstained with Hematoxylin. B. Immunohistochemistry analysis of Ring1B, S100A4 and Bmi1 expression in serial sections of two different invasive ductal breast carcinoma samples. Bars, 100 μm.

We analyzed Ring1B expression by immunohistochemistry in ten surgical samples of invasive ductal carcinoma (IDC) and in a commercial tissue microarray (TMA) consisting of six breast invasive ductal carcinoma tissues. Whereas Ring1B is barely detectable in the mammary ducts in histologically normal regions adjacent to the tumor, carcinoma cells display a moderate Ring1B staining signal (0.5 [0-1] and 2 [1-2.5], respectively; Spearman's rho correlation coefficient, 0.854; p<0.000). Interestingly, Ring1B expression reaches the maximal intensity of the immunohistochemistry staining (value of 3) in those cells invading the surrounding fat tissue in six out of the ten surgical IDC samples tested (Figure 1A). To better characterize these tumors, we performed immunohistochemistry to detect the expression of the cytoskeletal calcium-binding protein S100A4, since its nuclear, but not cytoplasmic, expression is associated with aggressive behavior of different epithelial tumors and poor patient outcome [25,26]. Indeed, nuclear S100A4 positive staining can be detected in tumoral cells invading the stroma (Figure 1B). Immunohistochemistry on sequential sections from the same tumor revealed that Ring1B expression is enhanced in those regions that displayed positive nuclear staining for S100A4 (Figure 1B), suggesting that Ring1B expression could be linked to a poor IDC prognosis. Since PRC1 exhibits a variable composition of proteins we also investigated Bmi1, that has been found overexpressed in breast cancer, where it is associated with a good prognosis [4]. In stark contrast to the enhanced expression of Ring1B in the cells invading the stroma, Bmi1 expression is maintained, or in many instances reduced, in these invading cells when compared to the expression in carcinoma cells inside the bulk of the tumor (Figure 1B), suggesting a functional difference between both PRC1 proteins in ductal breast cancer.

### Ring1B is coexpressed with Fak in IDC and is able to induce Fak expression

**Figure 2:**
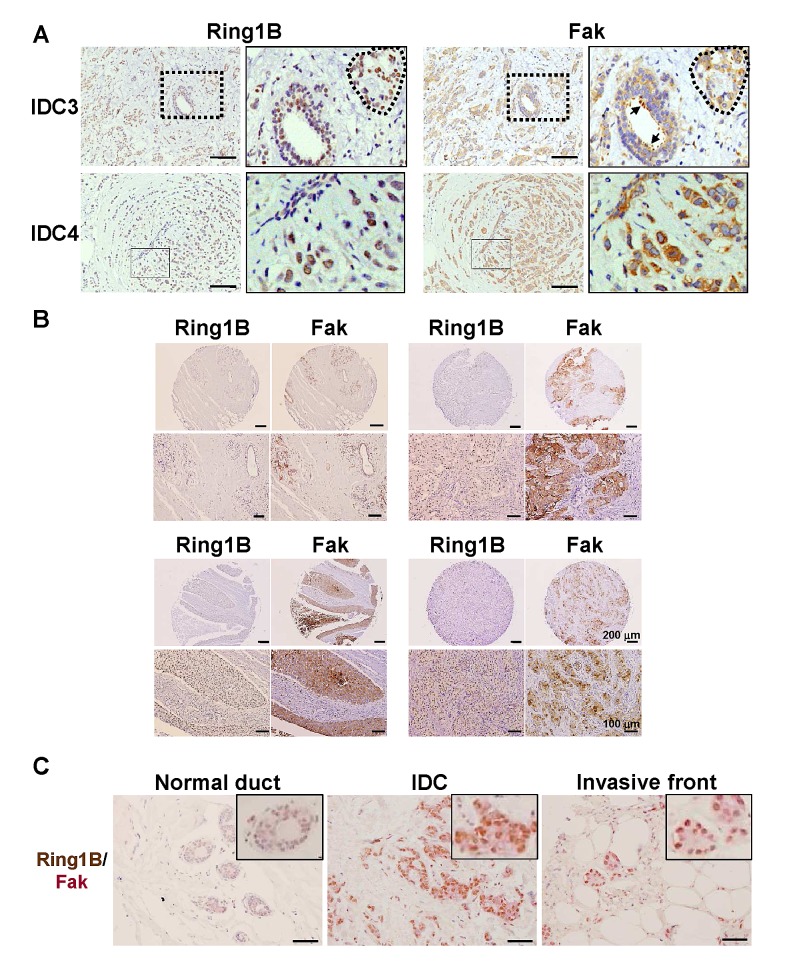
Ring1B expression is directly associated with Fak expression in invasive ductal carcinoma A. Immunohistochemistry analysis of Ring1B and Fak expression in serial sections of two invasive ductal breast carcinoma samples. On the left, higher magnifications of the fields indicated in the right panels. Dashed lines indicate carcinoma cells embedded in the tumoral stroma and arrows correspond to luminal cells of a duct within tumoral stroma. Observe that Ring1B and Fak expression patterns overlapped. Bars, 100 μm. B. Ring1B and Fak expression in serial sections of normal adjacent tissue (upper left corner) and three invasive ductal breast carcinoma tissues in a commercial tissue microarray. Lower panels, higher magnification pictures for the tumors shown above. C. Double staining analysis of Ring1B (brown) and Fak (pink) in adjacent normal mammary ducts and in carcinoma cells. Bars, 100 μm.

A key protein that integrates signals from growth factors and integrins to control cell migration and invasion is the non-receptor Focal adhesion kinase (Fak)[27]. Fak is required for the transition of premalignant hyperplasias to carcinomas and their subsequent metastases and a large proportion of primary human breast cancers possess elevated Fak expression that is further correlated with malignant transformation and poor clinical outcome [28,29]. Therefore, we investigated Ring1B and Fak expression in serial sections of IDC surgical samples. Basal cells of the ducts do not express Ring1B nor Fak, whereas luminal cells express both proteins. In addition, tumoral cells embed in the stroma display a positive staining for both Ring1B and Fak (Figure 2A). In the TMA tissues, a moderate to strong immunoreactivity for Fak can be detected in four of these tumors (4/6). Interestingly, Ring1B is expressed in three of these Fak-positive tumors (Figure 2B), whereas two tumors were negative for both Fak and Ring1B expression. To better characterize Ring1B and Fak relationship, we quantified Fak expression in tissue adjacent to the tumor and in tumoral cells of the surgical and the TMA samples. Whereas Fak is barely detectable in normal ducts, its expression in tumoral cells is enhanced (2 [2-3] *versus* 0 [0-1]). Correlation analysis revealed that the expression of Ring1B and Fak is associated both in normal adjacent ducts (correlation coefficient: 0.600, p<0.039) and in the IDC tumoral cells (correlation coefficient: 0.505, p<0.046). Double immunohistochemical staining on the same slides showed that IDC cells coexpress Ring1B and Fak (Figure 2C), further strengthening the link between Ring1B and Fak.

**Figure 3:**
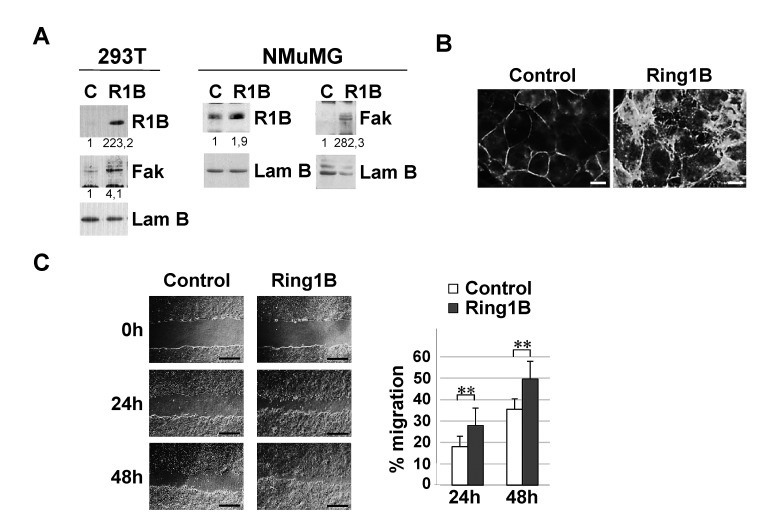
Ectopic Ring1B is able to induce Fak expression and cell migration A. Ectopically expressed Ring1B enhances Fak expression, as determined by western blot. Lamin B, loading control. Numbers at the bottom of each western blot lane represent protein band intensities normalized to Lamin B and relative to control cells, as described in “Methods”. B. Focal contact formation in Ring1B overexpressing NMuMG cells, determined by immunofluorescence to detect Fak. Bars, 10 μm. C. Ectopic Ring1B expression results in NMuMG cell migration, determined by scratch assays. Graph shows data (mean ± SD) from a wound healing assay of NMuMG cells transfected with empty or Ring1B expression vectors. Bars, 2 mM. Experiment was performed in triplicate and repeated three times with similar results. **, P < 0.005.

These observations lead us to investigate Ring1B ability to induce Fak expression in 293T cells, a cell line previously used to study Fak regulation by different transcription factors [30,31]. As Figure 3A shows, transient transfection of Ring1B results in the upregulation of Fak expression. Similar results were achieved when mammary epithelial NMuMG cells were transiently transfected with a Ring1B expressing vector. In these cells, ectopic Ring1B enhances Fak expression (Figure 3A) which localizes at focal contacts in the periphery of the cells (Figure 3B). In addition, Ring1B overexpressing NMuMG cells display enhanced cell migration, as determined by scratch assays (Figure 3C).

### Endogenous Ring1B sustains steady state Fak levels and is required for Tgfβ-induced phenotypic changes

**Figure 4:**
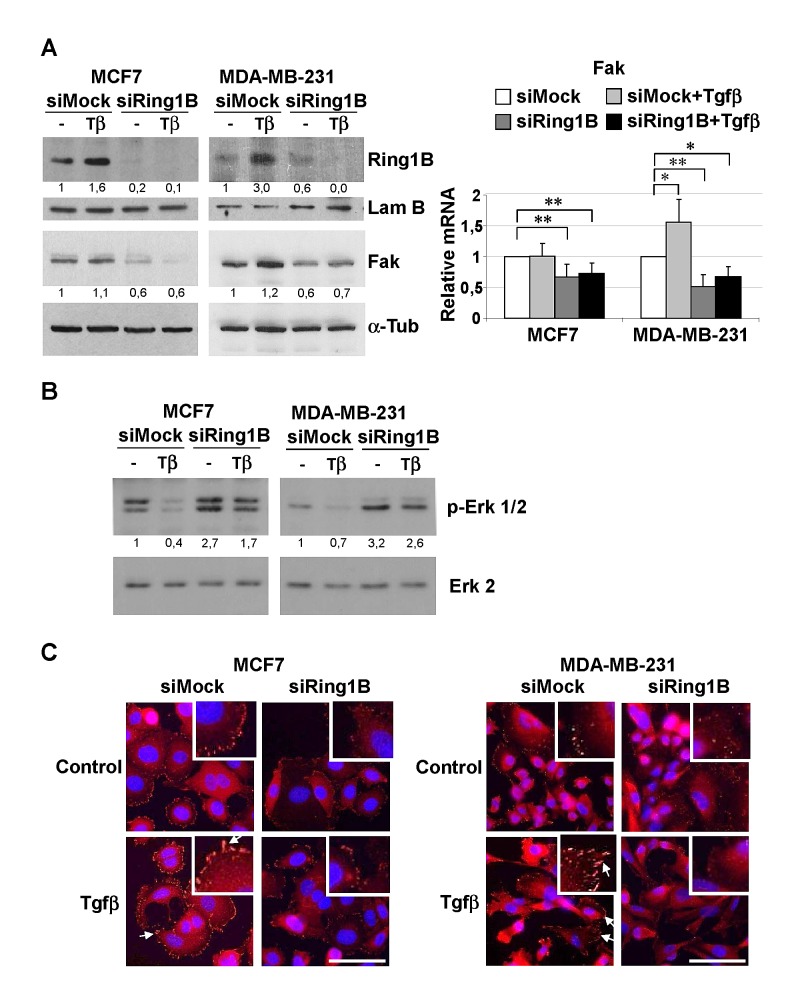
Endogenous Ring1B is required to sustain steady state levels of Fak, modulate Erk phosphorylation and to allow Fak recruitment to focal adhesions upon Tgfβ treatment in mammary cancer cell lines A. Cells were oligofected with mock or Ring1B siRNA and 24 hours later treated with 2 ng/ml Tgfβ. 48 h after transfection, protein and mRNA levels of Fak were determined by western blot and qRT-PCR, respectively. Effectiveness of Ring1B downregulation was confirmed by western blot (upper panel) and qRT-PCR (Supplementary Figure 1). α-Tubulin and Lamin B, loading controls. *, P < 0.05; **, P < 0.005. B. Erk phosphorylation in Ring1B-depleted cells upon Tgfβ treatment. MCF7 and MDA-MB-231 cells were treated as above and 48 hours later p-Erk 1/2 levels were determined by western blot. Erk 2, loading control. C. Subcellular localization of Fak in Ring1B depleted cells upon Tgfβ treatment, monitored by immunofluorescence. Arrows indicate focal adhesions. Bar, 75 μm.

To investigate the role of endogenous Ring1B in the expression of Fak in breast cancer cells, we downregulated Ring1B levels in the MCF7 and MDA-MB-231 cell lines by transfecting them with small interfering RNA oligos (siRNA). Knockdown efficiency was assessed by western blot and qRT-PCR (Figure 4A and Suppl. Figure 1, respectively). Since it has been reported that Tgfβ can induce Fak expression and activation [32,33], we also tested whether endogenous Ring1B is required to regulate Fak both in basal and in Tgfβ-treated cells. Ring1B depletion leads to drops in the steady state levels of Fak protein and mRNA, as revealed by western blot and qRT-PCR analysis respectively (Figure 4A). In addition, Tgfβ treatment does not restore Fak levels in Ring1B-depleted cells (Figure 4A) and Tgfβ-induced Y861-Fak phosphorylation is impaired in these cells (Suppl. Figure 2).

Fak tyrosine phosphorylation occurs at six or more sites *in vivo* and phosphorylation at the C-terminal Tyr 861 and 925 creates binding sites for the src homology 2 (SH2)-containing proteins, which are thus likely to participate in the regulation of downstream targets such as the Erk/MAP kinase cascade [32]. We therefore investigated Erk phosphorylation in control and Ring1B-depleted cells. Tgfβ treatment for 24 hours results in depression to below basal levels of Erk phosphorylation in control cells, whereas Erk phosphorylation is barely affected by Tgfβ treatment in Ring1B depleted cells (Figure 4B). In contrast, both control and Ring1B depleted cells are sensitive to the strong activator of the Erk signaling pathway TPA (Suppl. Figure 3), suggesting that the observed differences in Erk regulation upon Tgfβ treatment are due to a defective Fak pathway upstream of Erk in Ring1B depleted cells.

Next we determined whether Ring1B modulation impinge on Fak subcellular localization. Whereas some discrete punctate focal complexes at the cell periphery and a diffuse staining in the cytoplasm can be observed in basal conditions, Tgfβ treatment induces Fak translocation to newly formed focal adhesions (Figure 4C). In contrast, Ring1B depletion prevents Fak accumulation at focal adhesion complexes in basal and Tgfβ conditions (Figure 4C).

**Figure 5:**
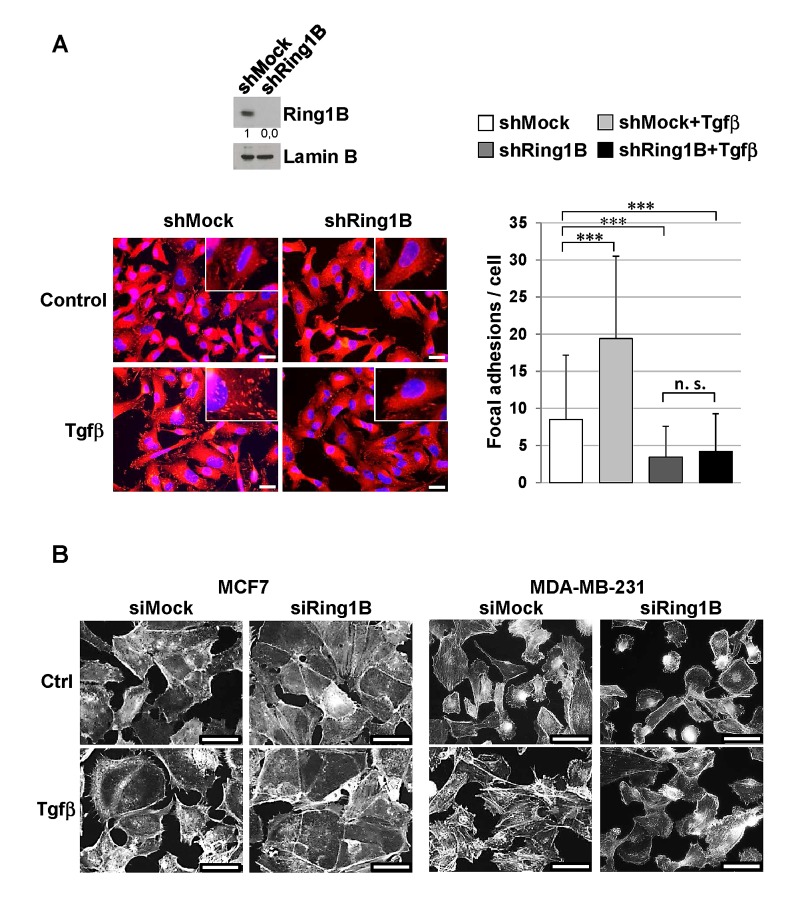
Endogenous Ring1B is required to allow Tgfβ-induced focal adhesion formation and phenotypic changes in mammary cancer cell lines A. Upper panel, efficiency of stable Ring1B knockdown (short hairpin, sh) in MDA-MB-231 cells determined by western blot. Lower panel, Vinculin inmunostaining in shMock and shRing1B MDA-MB-231 cells untreated or treated with 2 ng/ml Tgfβ. Bars, 20 μm. On the right, quantification of Vinculin foci, performed with a 40× objective. For quantification, more than 150 cells were scored from at least eight random fields in each group and data are mean ± SD. ***, P < 0.001; n. s., non-significant. B. Cells were oligofected with mock or Ring1B siRNA and treated with 2 ng/ml Tgfβ. Fourty eight hours later cells were stained with Rhodamine-Phalloidin, which binds specifically to F-actins to visualize stress fibers, actin filaments and focal contacts at stress fiber termini. Note that Tgfβ causes loss of intercellular adhesions and projection of cytoplasmic protrusions from the cellular body in control cells. In contrast, Ring1B-depleted cells are defective in forming these Tgfβ-induced extensions and MCF7 cells are also defective in losing cell-to-cell contacts in response to Tgfβ treatment. Bar, 50 μm.

Fak regulates steady-state adhesive forces by modulating Vinculin recruitment to focal adhesions [34]. Therefore we examined Vinculin localization in MDA-MB-231 cells stably depleted of Ring1B (shRing1B) (Figure 5A, upper panel). Immunofluorescence analysis of control (shMock) and shRing1B MDA-MB-231 cells revealed that the number of focal adhesions is significant reduced in shRing1B cells when compared to shMock MDA-MB-231 cells. Furthermore, Tgfβ treatment significantly increases the number of Vinculin-containg focal adhesions in shMock cells, but does not affect the number of Vinculin focal adhesions in shRing1B cells (Figure 5A).

Since Vinculin is an F-actin binding protein and this interaction is critical to coordinate F-actin organization and focal adhesion dynamics at the leading edge [35], we next analyzed the role of Ring1B in Tgfβ induced-morphologic changes by cytoskeletal F-actin labeling with Rhodamine-Phalloidin. No obvious or very subtle changes in cell morphology can be observed in Ring1B-depleted cells in basal conditions (in the absence of Tgfβ) when compared to control cells, whereas Ring1B depletion prevents Tgfβ−dependent stress fiber and focal contact formation (Figure 5B). These data indicate that endogenous Ring1B is required to sustain steady state Fak levels required for activation upon Tgfβ stimulus in mammary tumoral cells.

### Ring1B is required for cell migration and invasion of breast cancer cells

**Figure 6:**
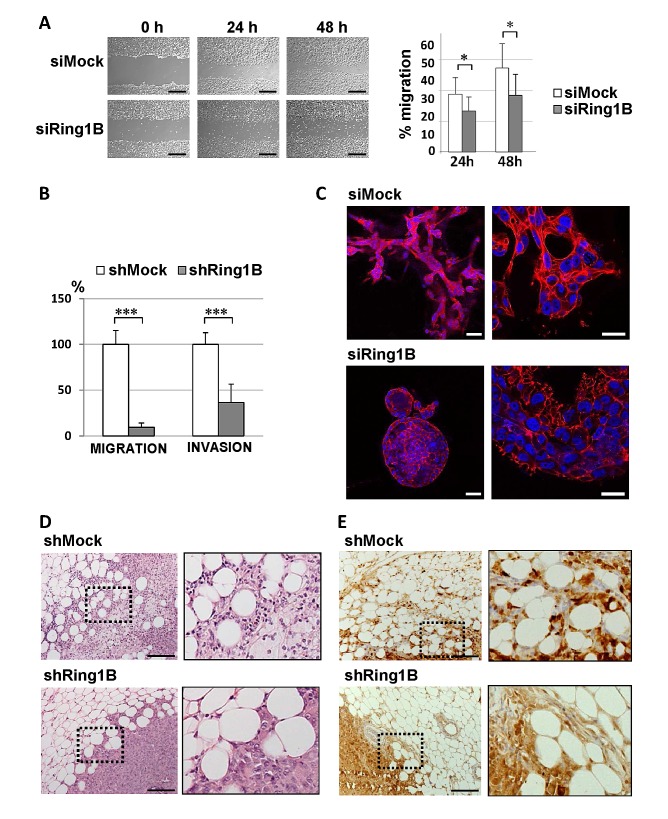
Ring1B is required for cell migration and invasion A. Wound healing assays of MCF7 cells oligofected with mock or Ring1B siRNA. Pictures were taken after 24 and 48 hours (left panel) and the area of the scratch remaining unfilled was quantified (right panel). Bars, 2 mM. Graph displays mean ± SD of three independent experiments performed in triplicate. *, P<0.05. B. Defective migration and invasion abilities of Ring1B-depleted MDA-MB-231 cells, analyzed by transwell assays. shRing1B migration and invasion are expressed as fold versus controls, shown as mean ± SD from two independent experiments performed in triplicate. ***, P < 0.001 C. Section of the colonies formed in Matrigel from siMock or siRing1B MDA-MB-231 cells, analyzed by Phalloidin staining (in red). DAPI was used for labeling cell nuclei (in blue). Bars, 50 and 25 μm (left and right pictures, respectively). D. Hematoxylin and Eosin staining of representative tumors from nude mice injected with control or Ring1B-depleted MDA-MB-231 cells (1x10^6^) into the mammary fat pad and sacrificed when their tumors reached 0.8 cm^3^. Bars, 100 μM. E. Immunohistochemistry detection of S100A4 in representative MDA-MB-231 orthotopic tumors. Bars, 100 μM. x20, original magnification. Right pictures, higher magnifications of the indicated fields.

We next assessed whether Ring1B deficiency functionally affects the mobility abilities of breast tumoral cells *in vitro*. Cell migration analysis performed by a scratch in the monolayer of confluent cultures showed that MCF7 control cells extensively migrate into the wounded area, whereas cell intrinsic migration is impaired in Ring1B-depleted cells (Figure 6A). We also examined the migratory and invasive abilities of shMock and shRing1B MDA-MB-231 cells by using transwell migration and invasion assays. As Figure 6B shows, Ring1B depletion significantly blocked cell migration and invasion in these cells. Next we tested whether this effect could be extended to other breast cancer cell lines. Indeed, MDA-MB-468, SUM159, BT549, SUM149 and SUM1315 cells transiently transfected with Ring1B siRNA display impaired abilities to migrate and to invade through basement membrane extract when compared to siMock control cells (Suppl. Figure 4). Furthermore, in three dimensional (3D) cultures, parental MDA-MB-231 cells invade the extracellular Matrigel matrix and F-actin accumulates mainly at the cell rear, whereas Ring1B-depleted MDA-MB-231 cells form acini-like spheroids with a hollow lumen (Figure 6C). Thus, Ring1B downregulation impairs the ability of mammary epithelial tumor cells to migrate and invade *in vitro*.

Next, we sought to determine whether Ring1B could be required for *in vivo* invasion by orthotopically injecting MDA-MB-231 cells stably depleted of Ring1B into mammary fat pads of female mice. Ring1B knockdown causes tumors that are histologically distinct from those originated by shMock MDA-MB-231 cells: whereas control tumors are poorly differentiated and clearly invade the surrounding adipose tissue, shRing1B tumors display polygonal cells that are unable to invade the adipose component (Figure 6D). To better characterize these tumors we performed immunohistochemistry to detect S100A4. Interestingly, three out of the five analyzed tumors formed by control MDA-MB-231 cells display intensity levels above 2 and mainly nuclear S100A4 staining, whereas S100A4 expression in all the tumors from Ring1B-depleted cells corresponds to an intensity of 1 and the staining is diffusely distributed (Figure 6E). Since Ring1B-depleted and control MDA-MB-231 cells proliferate in culture to a similar extent and form subcutaneous tumors of similar size (unpublished observations), these results indicate that endogenous Ring1B expression is necessary for regional spreading of the tumoral cells.

### Ring1B sustains Fak steady state levels by repressing p63

**Figure 7:**
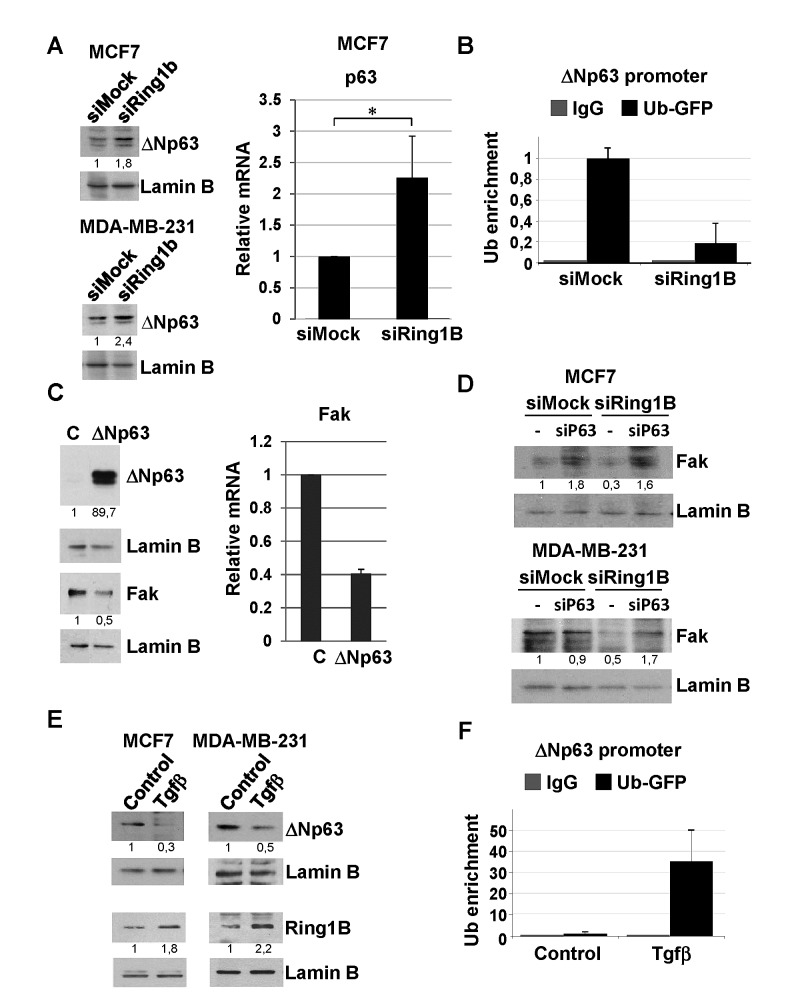
Identification of ∆Np63 as a molecular target involved in Ring1B-sustained Fak expression A. Analysis of ∆Np63 expression in cells depleted from Ring1B, detected by western blot analysis of nuclear extracts and qRT-PCR. *, P < 0.05. B. Cells depleted from Ring1B displayed an impoverishment of ∆Np63 promoter ubiquitination, as determined by chromatin immunoprecipitation (ChIP) of MCF7 cells transfected with GFP-tagged ubiquitin and oligofected with mock or Ring1B siRNA. Graph displays percentage of Ub-GFP recruitment to ∆Np63 promoter relative to input values and expressed as mean ± SD of two independent experiments. C. Ectopic ∆Np63 represses Fak expresion in transiently transfected MCF7 cells, as shown by western blot analysis and qRT-PCR. D. Effect of Ring1B dowregulation on Fak expression in cells oligofected with p63 siRNA, detected by western blot. E. Tgfβ represses ∆Np63 expression in MCF7 and MDA-MB-231 cells and induces ubiquitination of endogenous ∆Np63 promoter in MCF7 cells transiently transfected with GFP-tagged ubiquitin, as detected by ChIP. Graph displays percentage of GFP-ubiquitin recruitment to ∆Np63 promoter normalized to input values and expressed as mean ± SD of three replicate samples from one representative experiment out of two.

Next we attempted to identify the mechanism underlying the regulation of Fak expression by Ring1B in breast cancer cells. Ring1B is a histone ubiquitin-ligase mainly associated with gene silencing [36]. Therefore, the molecular mechanism involved in Ring1B-sustained steady state levels of Fak would likely be indirect via repression of a Fak inhibitor. According to this hypothesis Ring1B knockdown should result in the enhanced expression of a Fak repressor. Fak promoter contains p53 binding sites [30] and wild type, but not mutant, p53 binds to Fak promoter *in vivo* and inhibits Fak expression in cancer cells [37]. Therefore, we tested the possibility that Ring1B downregulation releases p53 expression, resulting in Fak repression. However, Ring1B depletion does not affect p53 protein levels neither in the p53-wild type MCF7 cells nor in the p53-mutant MDA-MB-231 cells (data not shown). A closely p53-related protein is p63, which can be transcribed from two different promoters, one that retains and another that lacks the transactivation domain (TA and ∆N, respectively) [38]. Since p63 DNA-binding domain is 60% identical to that of p53 and is able to interact with the consensus p53-responsive sequences [38,39], we checked whether p63 expression is affected by Ring1B depletion. Ring1B downregulation in MCF7 and MDA-MB-231 cells results in an enhancement of ∆Np63 expression, the isoform predominantly expressed in epithelial cells (Figure 7A). In addition, since Ring1B monoubiquitinates H2A, we also tested whether ubiquitination of ∆Np63 promoter is sensitive to Ring1B depletion. Indeed, GFP-ubiquitin-tagged cells transfected with Ring1B siRNA display reduced levels of GFP-ubiquitin at the ∆Np63 promoter, suggesting that this promoter is directly and actively repressed by Ring1B-mediated histone ubiquitination (Figure 7B). To investigate ∆Np63 ability to modulate Fak expression, MCF7 cell were transiently transfected with ∆Np63 expression vector. Indeed, ∆Np63 overexpression results in a decrease in Fak expression (Figure 7C), suggesting that release of ∆Np63 expression upon Ring1B depletion could be responsible for the observed Fak downregulation. To formally test this possibility we checked whether Ring1B knockdown affects Fak expression in p63 deficient cells. To this end, MCF7 and MDA-MB-231 cells were oligofected with p63 siRNA and the efficiency of the knockdown was evaluated by western blot and qRT-PCR (Suppl. Figure 5). In p63-deficient cells, Ring1B depletion does not result in diminished Fak levels (Figure 7D), suggesting that release of p63 expression is required to mediate Fak repression upon Ring1B downregulation.

Since we consistently observed that Tgfβ treatment moderately induces Ring1B expression, we wondered whether the cytokine could reinforce Ring1B-mediated ΔNp63 repression. Indeed, Tgfβ treated cells display lower levels of ΔNp63 (Figure 7E) and enhanced levels of GFP-ubiquitin at ΔNp63 promoter (Figure 7F), pointing to a direct role of Ring1B in Tgfβ-mediated ΔNp63 regulation.

## DISCUSSION

Metastasis is the main cause of death in cancer patients and is widely considered a multistep process initiated when cells originating from the primary tumor undergo several morphological changes, acquiring the ability to spread to distant organs. We had previously shown that Ring1B expression is overexpressed in high-grade pancreatic intraepithelial neoplasia and in pancreatic ductal adenocarcinoma [24]. Supporting Ring1B involvement in cancer progression, its strongest expression is found in those tumoral cells with nuclear expression of the metastasis-promoting protein S100A4 that invade the adipose tissue both in human IDC as well as in mammary gland xenografts. Importantly, in this experimental model, Ring1B depleted MDA-MB-231 cells fail to invade the surrounding mammary fat pad and display reduced S100A4 nuclear staining. These data suggest that endogenous Ring1B levels in tumoral cells of the IDC invading front could be regulated by growth factors or cytokines produced by the tumor or the tumoral stroma, such as Tgfβ.

Ring1B displays an overlapping expression pattern with Fak in IDC tumors and ectopic Ring1B in cell lines in culture results in Fak induction. Orthotopic and transgenic mouse models of breast cancer revealed that Fak signaling is essential during the initial steps of metastasis formation for the transition of premalignant hyperplasia to carcinoma and the subsequent metastases [29,40]. Furthermore, Fak protein has been shown to be elevated in tumour tissues [41] and the underlying genetic mechanisms include copy number gains, amplification and isochromosome formation involving the FAK locus [42]. Our results indicate that Ring1B is necessary to sustain Fak basal levels in breast tumor cell lines, for proper cell migration and invasion and for Tgfβ−dependent stress fiber and focal contact formation. Accordingly, Ring1B was shown to be essential in maintaining an undifferentiated state in stem cells by affecting ECM-related pathways that are known to be involved in the regulation of Fak phosphorylation as well [36]. Recent data demonstrate Ring1B ability to function as E3-ligase in the ubiquitination of proteins other than histones [43,44]. Therefore, Ring1B requirement for Fak activation could rely not only on mechanisms of transcriptional repression but can also be due to postranslational modifications of proteins involved in Tgfβ-signal transduction. Further efforts will be required to characterize other targets of Ring1B-mediated ubiquitination and whether any of them can have an impact on Fak function.

Ring1B depletion resulted in a decrease in Fak expression concomitant with an increase in ΔNp63 levels and to a drop in the ubiquitination degree of ΔNp63 promoter. Ectopic ΔNp63 is able to repress Fak expression, possibly by transcriptional and posttranscriptional mechanisms. Indeed, it has been reported that ΔNp63 modulates the expression of Hsp70, a chaperone that prevents caspase 3-mediated proteolysis of Fak [45]. In addition, Hsp70 overexpression is associated with metastasis and with short-term disease free survival metastasis and poor prognosis in breast cancer [46]. Therefore, Ring1B repression of ΔNp63 could be critical for the maintenance of Fak expression and activity in breast tumoral cells.

## CONCLUSIONS

The PcG protein Ring1B is overexpressed in invasive ductal breast carcinoma and its expression pattern in these tumors is coincidental with Fak expression pattern. Endogenous Ring1B sustains Fak steady state levels in breast cancer cells and is required for *in vitro* and *in vivo* migration and invasion. Therefore we propose that Ring1B should be considered as a new molecular marker for tumoral progression and as a putative therapeutic target.

## METHODS

### Human breast cancer samples

Ten human breast cancer tissues from the Pathology Department (Hospital del Mar) were evaluated by an expert pathologist (JMC) to classify the lesions. Procedures were approved by the Ethical Committee for Clinical Research of our institution (Institut Municipal d'Assistència Sanitària, Protocol # 2008/3275/I), waiving the requirement for informed consent for the study. A commercially available breast cancer tissue array (Biomax Inc., Rockville, MD, USA; cat. No. BR243f) including 6 cases of breast invasive ductal carcinoma was also analyzed by immunohistochemistry.

### Immunohistochemistry

Immunohistochemical analyses were performed using 5 μm sections of formalin-fixed, paraffin-embedded tissue blocks. Antigen retrieval was performed in 10 mM citrate (pH 6) for 15 min in a pressure cooker. The slides were then incubated with primary antibodies for 12 hours. After washing, the Envision^+^ System-HRP antibody reagent was applied (Dako, Glostrup, Denmark). Reactions were developed using diaminobenzidine (DAB) as chromogenic substrate. Sections were counterstained with hematoxylin, dehydrated and mounted. For sequential horseradish peroxidase/alkaline phosphatase (HRP/AP) immunoenzymatic double staining analysis of Ring1B and Fak, sections were first stained with primary anti-Ring1B, followed by Envision^+^ System-HRP antibody. Subsequently slides were washed extensively and incubated with anti-Fak primary antibody followed by incubation with AP-conjugated secondary antibody. Finally, HRP activity was developed as above and AP detection was performed by incubation with K1395 Fast Red reagent (Dako, Glostrup, Denmark). Ring1B, Bmi1 and Fak antibodies were purchased from Millipore (Billerica, MA, USA) and S100A4 antibody was from Abcam (Cambridge, MA, USA). AP-conjugated secondary antibody was purchased from Dako (Glostrup, Denmark).

Immunohistochemical staining was scored by two observers (AB and IHM). The intensity (0, 1, 2, 3) of the immunostaining was evaluated and scored as follows: 0, negative or trace amounts; 1, low staining; 2, medium staining; 3, strong staining. Results are expressed as mean [confidence interval] and statistical analysis was performed with the Spearman's rho.

### Cell culture conditions

Cells were grown in DMEM or DMEM:F12 (Gibco, Life Technologies, Paisley, UK) in standard conditions. To perform Tgfβ treatments, cells were treated with 2 ng/ml Tgfβ (Peprotech, Rocky Hill, NJ, USA) for 24 hours. TPA (12-O-tetradecanoylphorbol-13-acetate) was purchased from Sigma (St. Louis, MO, USA) and cells were treated with 200 nM TPA for 24 hours or 15 minutes.

### Oligofection, transfection and retroviral infection

Oligofection has been performed as described earlier [24]. To generate retroviral stocks, Phoenix cells were transfected with Fugene (Roche, Indianapolis, IN, USA). For stable expression of Ring1B short hairpin RNA (shRNA), cells were transduced with retroviral supernatants in the presence of Polybrene (4 μg/ml; Sigma, St. Louis, MO, USA) and selected with 2 μg/ml Puromycin (Sigma, St. Louis, MO, USA). For transient expression of GFP-ubiquitin, ΔNp63 or Ring1B cells were transfected with Lipofectamine 2000 (Invitrogen, Life Technologies, Paisley, UK). Expression vector for Ring1B was generated by using standard PCR methods and verified by sequencing. GFP-ubiquitin expression vector was obtained from Addgene (plasmid 11928)[47]. ΔNp63 expression vector was a generous gift from Dr. Caron de Fromentel (INSERM U590, Lyon, France). Retroviral constructs carrying shRNA sequences with Puromycin resistance were essentially made as previously described [48]. Mock, Ring1B and p63 siRNA sequences are available upon request.

### Protein extraction, western blot and immunofluorescence

These protocols have been performed following standard techniques. Ring1B, Fak and p63 antibodies were purchased from Millipore (Billerica, MA, USA). pY861-Fak antibody was from Biosource International (Life Technologies, Paisley, UK). Anti-activated MAP kinase (diphosphorylated Erk 1/2) was from Sigma (St. Louis, MO, USA) and Erk 2 (C-14) antibody was purchased from Santa Cruz Biotechnology (Heidelberg, Germany). Lamin B1 antibody was from Abcam (Cambridge, MA, USA) and Vinculin and α-Tubulin antibodies from Sigma (St. Louis, MO, USA). Fluorescent secondary antibodies were obtained from Invitrogen (Life Technologies, Paisley, UK) and Jackson ImmunoResearch Laboratories (West Grove, PA, USA). Rhodamine-Phalloidin was from Sigma (St. Louis, MO, USA). For focal contact counting, the cells were labeled with anti-Vinculin antibody and the Vinculin-positive patches were manually counted.

Western blot quantification was performed using Image J densitometry software. The intensity of individual bands was normalized to Lamin B or α-Tubulin signal, as a measure of protein relative abundance in the different samples and referred to control conditions.

### RNA isolation and qRT-PCR

RNA was isolated with Genelute Total Mammalian RNA Kit (Sigma, St. Louis, MO, USA) and cDNA was obtained by using Transcriptor First Strand cDNA Synthesis Kit (Roche, Indianapolis, IN, USA). qRT-PCR assays were performed using SYBR Green PCR master mix (Applied Biosystems, Life Technologies, Paisley, UK)). For normalization purposes, we run simultaneously qRT-PCR with primers for Gapdh. The ABI PRISM 7900HT cycler's software calculated a threshold cycle number (Ct) at which each PCR amplification reached a significant threshold level. Figures present the amount of target mRNA/Gapdh mRNA relative copies ratio. Primers used for qRT-PCR were: Ring1B (5'-CAGACAAACGGAACTCAACCATT-3'; 5'-CTGTTATTGCCTCCTGAGGTGTT-3'), Fak (5'-GCAGTCATTTATCATCAGACCTCAGA-3'; 5'-GCCTTGCTTTTCGCTGTTG-3'); p63 (5'-CTTGCCCAGGAAGAGACAGG-3'; 5'-CATAAGTCTCACGGCCCCTC-3'); Gapdh (5'-AGTCAGCCGCATCTTCTTTTG-3'; 5'-AAATCCGTTGACTCCGACCTT-3').

### Chromatin immunoprecipitation assays (ChIP)

GFP-ubiquitin tagged cells were cross-linked with 1 % formaldehyde for 10 min and lysed in buffer IP1 (10 mM Hepes-KOH pH 7.5, 10 mM NaCl, 3 mM CaCl_2_, 0.25 M sucrose, 1 mM DTT, 1 mM PMSF). After 30 min on ice, nuclei were collected by centrifugation at 3000 g for 10 min. The pellet was lysed in IP2 buffer (50 mM Tris pH 8.0, 10 mM EDTA, 1% SDS) and sonication 10 times at 40 % 10 sec (Branson). Immunoprecipitation was carried out with anti-GFP antibody (Clontech, Mountain View, CA, USA) or irrelevant immunoglobulin G (Sigma, St. Louis, MO, USA) in IP buffer (16.7 mM Tris pH 8.0, 167 mM NaCl, 1.2 mM EDTA, 1.1 % Triton X-100, 0.01 % SDS). Samples were then processed as indicated in EZ ChIP™ Kit (Upstate, Cell Signaling Technology, Danvers, MA, USA). DNA was purified using GFX PCR DNA purification kit (Amersham, GE Healthcare Europe GmbH, Freiburg Germany). Promoter regions were detected by qPCR with SYBR Green PCR master mix (Applied Biosystems, Life Technologies, Paisley, UK). ChIP results were quantified relative to the input amount. Chromatin immunoprecipitation primers for human ΔNp63 promoter were 5'-GGTTGGCAAAATCCTGGA-3'; 5'-TCACTAAATTGAGTCTGGGCATT-3'.

### Wound healing assay, transwell migration and invasion, 3D cultures and xenograft assays

Cells previously oligofected or transfected were seeded in standard conditions. 24 h later they were incubated with 10 μg/ml Mitomycin C (Sigma, St. Louis, MO, USA) for 1 h and confluent monolayers were scratched with a plastic pipette tip. Healing was measured at 0, 24 and 48 hours by using Image J software and expressed as a percentage of the area of the scratch remaining unfilled.

Cell migration assays carried out in transwell chambers were performed as follows: cells were harvested with trypsin, washed with PBS and resuspended in serum-free DMEM. Cells were placed in the upper compartment of a Transwell 96 well permeable supports (1x10^5^ cells in 100 μl / well). The lower compartment was filled with DMEM supplemented with 10 % fetal bovine serum (FBS). The two compartments of the chambers were separated by a 8.0 μm pore size PET (polyester) membrane (Corning Costar, Acton, MA). Chambers were incubated at 37 °C in 5% CO_2_ for 24 h. At the end of the incubation period, cells remaining on the upper surface were mechanically removed and cells on the lower chamber were incubated 4 h with hexosaminidase substate [49]. Thereafter, developer solution was added to each well and absorbance at 410 nm was recorded with a fluorescent reader Infinite M200 (TECAN) [49]. For cell invasion assays, PET membranes of the Transwell 96 permeable supports were coated with Basement Membrane Extract (Cultrex, AbBcn, Barcelona, Spain) and cells were processed as above. Assays were performed in triplicate and repeated two times.

3D cultures were performed as described previously [50]. In brief, 3x10^3^ MDA-MB-231 oligofected cells were seeded onto Matrigel beds (BD Bioscience, Franklin Lakes, NJ, USA) in 8-well culture slides (BD Bioscience, Franklin Lakes, NJ, USA) and maintained in growth medium supplemented with 2 % Matrigel. Change of medium was performed every 48 hours for 12 days with additional oligofections at days 4 and 8 to maintain the knockdown. Cells were fixed on 4 % formaldehyde at day 12 and stained with Texas Red-X Phalloidin (Invitrogen Life Technologies, Paisley, UK). Images were taken using a Leica TCS SP5 DMI microscope (Barcelona, Spain).

Orthotopic xenograft assays were performed as previously described [51]. Briefly, MDA-MB-231 cells were injected (1x10^6^ in 0.05 mL serum-free growth medium) into the mammary fat pads of female athymic Nude mice aged 8 weeks (Charles River, Barcelona, Spain). Tumor growth was measured every 2 days and when they reached a size of 0.8 cm^3^, they were surgically excised. A minimum of 10 tumors from each condition were generated and, at least 4 different tumors derived from each condition were analyzed. Mice were used in accordance with institutional guidelines approved by the Use Committee for Animal Care.

### Statistical Analysis

Each experiment was performed in triplicate at least three times unless otherwise indicated. Statistical analysis was performed with Student's *t*-test. *P* < 0.05 was considered significant.

## SUPPLEMENTARY FIGURES


