# Supplementary material for: The Polycomb group protein RING1B is overexpressed in ductal breast carcinoma and is required to sustain FAK steady state levels in breast cancer epithelial cells
Source: Oncotarget. 2014 Feb 14;5(8):2065–76. doi: 10.18632/oncotarget.1779 (PMC4039145; doi:10.18632/oncotarget.1779)
Supplement: Supplementary file 1 [file oncotarget-05-2065-s001.pdf]

The Polycomb group protein RING1B is overexpressed in ductal breast carcinoma and is required to sustain FAK steady state levels in breast cancer epithelial cells – Bosch et al

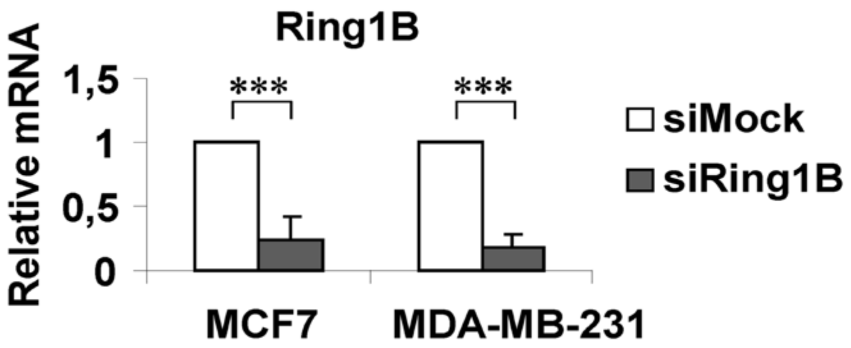

Supplementary Figure 1: Efficiency of Ring1B depletion, determined by qRT-PCR. ***, P<0.001.

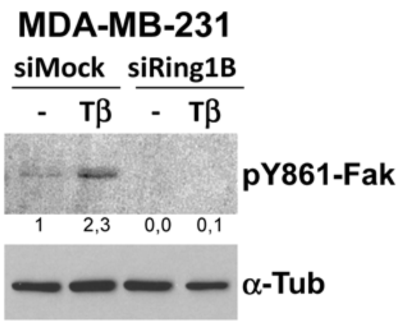

Supplementary Figure 2: Fak phosphorylation in Ring1B-depleted cells upon Tgfβ treatment. Parental and Ring1B-depleted MDA-MB-231 cells were treated with 2 ng/ml Tgfβ and 48 hours later pY861-Fak levels were determined by western blot.

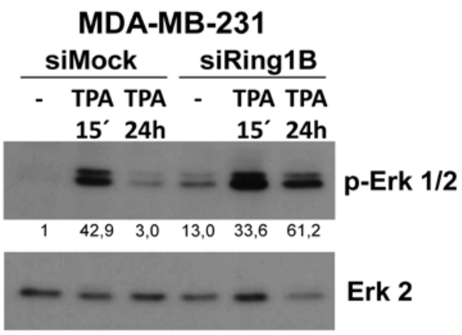

Supplementary Figure 3: Erk phosphorylation in control and Ring1B-depleted cells upon TPA treatment. Cells were oligofected with mock or Ring1B siRNA and 24 hours later treated with 200 nM TPA for 15 minutes or 24 hours. p-Erk 1/2 levels levels were determined by western blot. Erk 2, loading control.

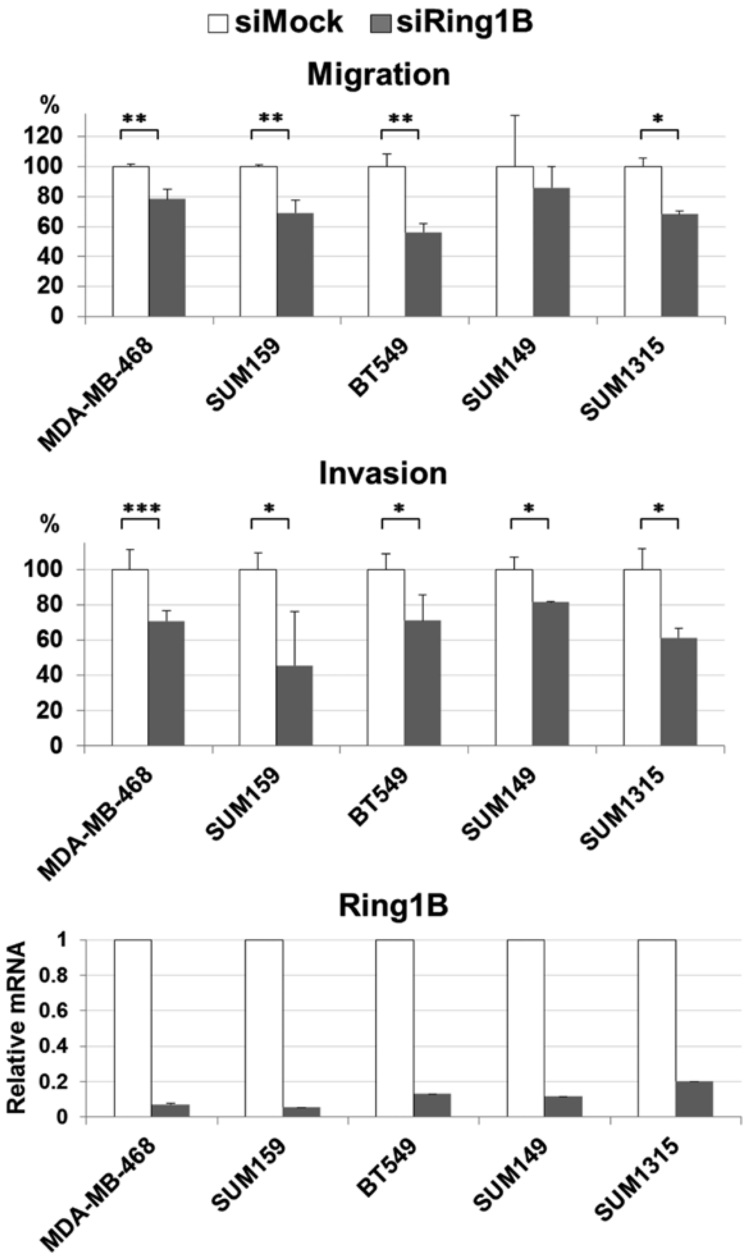

Supplementary Figure 4: Impaired ability of MDA-MB-468, SUM159, BT549, SUM149 and SUM1315 breast cancer cells to migrate and to invade through basement membrane extract. siMock and siRing1B cells were plated in permeable supports or in basement membrane extract coated chambers and allowed to migrate or invade, respectively, toward DMEM containing 10% FBS as a chemoattractant for 24 hours. siRing1B migration or invasion is expressed as fold versus controls and shown as mean ± SD from two independent experiments performed in triplicate. *, $P<0.05$; **, $P<0.005$; ***, $P<0.001$. Lower panel, efficiency of Ring1B depletion, determined by qRT-PCR.

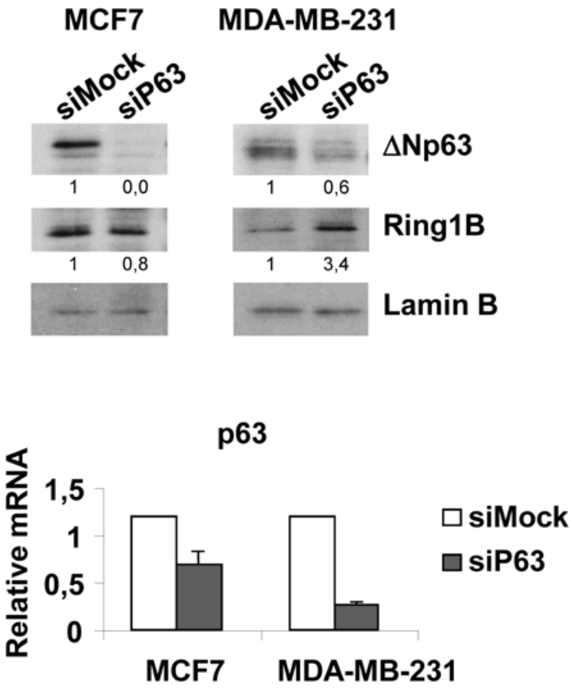

Supplementary Figure 5: Efficiency of p63 depletion, determined by western blot and qRT-PCR.
